# The health literacy level and its related factors in Iranian women with breast cancer undergoing chemotherapy

**DOI:** 10.3389/fpubh.2023.1150148

**Published:** 2023-09-28

**Authors:** Reyhaneh Sadeghian, Mahsa Asadollahi Hamedani, Sajad Salehipour, Anahita Sarabandi, Fatemeh Kiani, Hassan Babamohamadi

**Affiliations:** ^1^Department of Nursing and Midwifery, Hormozgan University of Medical Sciences, Bandar Abbas, Hormozgan, Iran; ^2^Department of Nursing, School of Nursing and Midwifery, Semnan University of Medical Sciences, Semnan, Iran; ^3^Department of Dentistry, Zahedan University of Medical Sciences, Zahedan, Iran; ^4^Department of Nursing and Midwifery, Zahedan University of Medical Sciences, Zahedan, Iran; ^5^Nursing Care Research Center, Semnan University of Medical Sciences, Semnan, Iran

**Keywords:** breast cancer, chemotherapy, health literacy, women, Iran

## Abstract

**Background:**

Breast Cancer (BC) is the most common cause of female mortality throughout the world. Promoting public awareness about this disease is the most crucial method of its prevention or control. The present study was carried out to determine the health literacy level and its related factors in women with BC.

**Methods:**

This cross-sectional study was conducted on BC patients undergoing chemotherapy in two teaching hospitals affiliated to Zahedan University of Medical Sciences in 2020. A total of 210 patients referred to these chemotherapy centers were selected by purposive sampling. The data collection tools included a demographic information form and a health literacy questionnaire for Iranian women with BC. The collected data were analyzed using descriptive and inferential statistics (logistic regression) in SPSS-22. *p*-values less than 0.05 were considered statistically significant.

**Results:**

The mean total score of the health literacy of women with BC undergoing chemotherapy was 40.35 ± 19.01, which suggests an insufficient health literacy. The health literacy had a significant relationship with variables including university education (OR = 4.41, *p* = 0.005) and supplementary insurance coverage (OR = 5.83, *p* < 0.001).

**Conclusion:**

The findings showed that university education and supplementary insurance coverage are associated with a higher health literacy among women with BC. Improving these factors and paying further attention to their role in the promotion of health literacy can help enhance the health literacy of women with BC.

## Introduction

Currently, cancer is one of the leading causes of death worldwide (1). More precisely, it is the second leading cause of global mortality and the most prevalent cause of death of women aged 35–55 years old (2), especially those with low socioeconomic status (3). According to the World Health Organization (WHO), one in eight women develops Breast Cancer (BC) (2). In 2020 in the United States, about 276,480 cases of BC and 42,170 deaths following BC have been recorded in women (4–6). According to the latest statistics in Iran, one in ten women is likely at risk of BC and the incidence age of BC in Iranian women is at least a decade less than that of women in developed countries. The average diagnosis age of BC in western countries is 56 versus 45 years in Iran (2). Also, 76% of cases of cancer among Iranian women are ascribed to BC (7).

Based on the literature, female gender and age are two significant risk factors of BC (8, 9). In addition, family history, alcohol use, menstruation before age 12 and menopause after 55 years, obesity, childbirth after age 30 years, breastfeeding withdrawal, certain medications such as diethylstilbestrol, large breast size and taking hormones increase the chance of developing BC (10).

BC treatment methods depend on the tumor type and stage as well as the patient’s clinical factors, including surgery, radiotherapy and chemotherapy alone or a combination of these methods (10). In most cases, BC remains undiagnosed until advanced stages, and when diagnosed, these therapeutic measures are no longer effective and only palliative care is used to improve the patient’s quality of life (10, 11). With the growing prevalence of BC, societies incur economic and social costs, and the role of early diagnosis and prevention of the disease in cost reduction becomes evident (12). BC can be prevented or diagnosed by screening methods such as mammography, breast self-exam (BSE) and clinical examination by a specialist (5).

By early screening, most BCs can be diagnosed at their primary stages before the patient shows any symptoms or the disease progresses (10). This observation demonstrates the importance of health literacy for the prevention or timely treatment of this disease (13). Health literacy is a broad concept that has been introduced by the WHO as an important determinant of health (14). The numerous definitions available for health literacy show that this concept has several dimensions in general: Capacity and accessibility, perception, processing and evaluation, decision-making and behavior regarding information, medical services and health (5). Various factors affect health literacy, including culture and ethnicity, family and social and cultural factors. Research has shown that limited health literacy (13) is associated with older age, lower income, and less years of formal education (15).

Health literacy and adverse health outcomes are negatively correlated, and the weaker is one’s knowledge about health conditions, the less will be her use of preventive strategies. Prolonged hospital stay, overuse of emergency services, overuse of medications, poor information about the uses of medications, inability to read and understand medication labels and health messages, and poor reporting of health status are the consequences of limited health literacy that affect not only the patient but also her family and the treatment system (16, 17). Other negative consequences include reduced screening, prolonged diagnostic phase, reduced acceptance and adherence to treatment, and less participation in clinical trials (18). Since the WHO identifies health literacy as one of the most significant determinants of health, its absence is a predictor of negative consequences in patients, such as cancer patients (4, 18).

Cancer treatment is a stressful life experience, and for this reason, patients diagnosed with cancer need information to better understand the disease, make decisions and receive appropriate treatment. Treatment is the most important part after getting a cancer diagnosis that enables the patient to return to her daily life and be able to fulfill her personal, social and family roles (19). The level of awareness and knowledge of patients undergoing chemotherapy plays an important role in their self-care and satisfaction with life (20). The lack of knowledge about chemotherapy and how to manage its side effects can lead to increased hospitalization and complications and reduce the patient’s quality of life (21). Today, it is known that the lack of adequate health knowledge among cancer patients will have detrimental effects (22, 23).

Given the increasing prevalence of BC and its associated mortality, investing on increasing the knowledge of Iranian women combatting this cancer can help with its early diagnosis and reduction of the disease complications (24). According to the results of national and international reviews, few studies have assessed these patients’ health literacy. Experts agree that implementing a range of strategies may be the most effective method of overcoming limited health literacies (25).

In a study conducted by Ahmadzadeh et al. (26) on improving self-care management in patients with BC through health literacy promotion, a direct and significant association was observed between health literacy and self-care dimensions in these patients, and enhancing their health literacy resulted in improved self-care. In a study conducted by Wei et al. (27) on the association between health literacy and quality of life in the survivors of BC, the results showed that health literacy has a significant effect on health-related quality of life (HRQOL), and as the patients’ quality of life and self-care behaviors improved, their disease prognosis was also ameliorated. Another study conducted in 2021 by Kanu et al. on cancer health literacy and patient activation in those with BC and their association with HRQOL showed that interventions aimed to improve HRQOL in patients with BC should target modifiable factors such as patient activation. In the cited study, the homogeneity of cancer health literacy among the participants might have influenced the non-significance of the association between HRQOL and patient activation (28).

To the best of the researchers’ knowledge, few studies have evaluated health literacy and its related factors in cancer patients. Therefore, assessing the health literacy of cancer patients, especially BC, helps the nursing community provide more effective and efficient care. On the one hand, by recognizing the existing challenges and understanding the gaps, nurses can apply strategies to improve them, and on the other hand, by recognizing the factors related to the health literacy level, more successful preventive programs can be implemented in the society. Therefore, the present study was conducted to determine health literacy level and its related factors in women with BC undergoing chemotherapy in teaching hospitals affiliated to Zahedan University of Medical Sciences.

## Materials and methods

### Design, setting, and participants

The present cross-sectional study was performed on all women with BC referred to the chemotherapy centers of two hospitals affiliated to Zahedan University of Medical Sciences, located in the south of Iran, from June to January 2020. A total of 210 women with BC who met the inclusion criteria were selected and enrolled in the study by census sampling, and their data were ultimately analyzed. The response rate was 100%.

The inclusion criteria were completing the informed consent to participate in the study, age ≥ 18 years, ability to communicate effectively, and having undergone at least one chemotherapy session. The exclusion criteria were the patient’s reluctance to continue participation in the study and developing other cancers. Considering the 95% confidence interval and 90% statistical power, the sample was estimated at about 103 patients based on the study by Rakhshkhorshid et al. (5). Nonetheless, since this study sought to examine the effect of demographic variables on women’s health literacy and in order to ensure sample size adequacy and take account of potential attrition, 107 extra patients were added to the samples and the present study was ultimately conducted on 210 patients.

### Data collection and instruments

The data collection tools included a demographic information form with items on age, marital status, education, etc., and a questionnaire about the health literacy of Iranian women with BC which was developed and psychometrically assessed by Khalili et al. (24). To evaluate the validity of the study tool, three methods were used, including content, face and structural validity assessments. Face validity was assessed using both qualitative and quantitative methods, which explained a total of 64.98% of the data dispersion, and Cronbach’s alpha coefficient was higher than 70% for all the parts (24). The tool reliability was calculated as 96.7%, which is considered acceptable.

The health literacy questionnaire used in this study had 34 items in five dimensions, including reading (items 1–4), access (items 5–11), understanding and perception (items 12–20), evaluation and judgment (items 21–25), and decision-making and behavior (items 26–34). The women scored themselves in each dimension of health literacy based on a 5-point Likert scale (quite easy = 5, easy = 4, neither easy nor difficult = 3, hard = 2, quite hard = 1). The respondent’s health literacy was calculated as the total points earned in each dimension. The raw score of the subscales equaled the algebraic sum of the scores for each respondent. The following equation was then used to convert this score into a 0-100 range:

${\left[\begin{array}{l} \left(\mathrm{Calculated}\;\mathrm{raw}\;\mathrm{score}-\mathrm{Minimum}\;\mathrm{raw}\;\mathrm{score}\right)/\\ \left(\mathrm{Maximum possible score}-\mathrm{Minimum possible score}\right) \end{array}\right]}^{*}\;100.$

The minimum score was 34 and the maximum 170, and scores of 0–50 indicated inadequate health literacy, 50.1–66 relatively inadequate health literacy, 66.1–84 sufficient health literacy and 84.1–100 excellent health literacy. The closer was the respondent’s score to 100, the higher was their health literacy (24).

### Ethical considerations

The principles of the World Medical Association Declaration of Helsinki apply to this study and the researchers ensured to respect participants’ rights and protect their health. This study also adheres to the ethical principles of research dictated by Zahedan University of Medical Sciences and received ethical approval from the university’s Research Ethics Committee (IR.ZAUMS.REC.1399.242). After getting a referral letter to be submitted to Imam Ali and Khatam al-Anbiya hospitals, the researchers visited the oncology wards of these hospitals and performed sampling until the desired sample size was reached. Since the current study was cross-sectional and the only risk was concerned with participants’ privacy, the researchers introduced themselves to the patients and provided sufficient explanations about the study objectives and methods, the confidentiality of the data, and the voluntary nature of participation in the study before beginning surveying. Written informed consent was obtained from patients who met the inclusion criteria and were willing to participate in this research project. The demographic information form and the health literacy questionnaire were individually read for the patients after chemotherapy and their answers were entered into the questionnaires.

### Data analysis

A statistical analysis of the data was performed using SPSS-22 at a significance level of 0.05. The categorical variables were expressed as frequency and relative frequency and the quantitative ones as mean and standard deviation (SD) for normally distributed data, and medians and interquartile ranges (IQR) for non-normally distributed data. The Kolmogorov–Smirnov test was used to determine the normal distribution of the data before beginning the analysis. Since the normality assumption was not met for the health literacy scores in the present sample, the outcome variable was divided into two categories based on the median of 41.9 points, and a fitted multiple logistic regression model was then used to examine the relationship between each of the predictor variables and the recent two-state variable and to estimate the odds ratio (OR). The final model was developed in three steps. First, the simple univariate models were fitted for each explanatory variable and the crude OR was obtained. A multiple model was then fitted in the presence of all the explanatory variables. In the third stage, a stepwise method and the likelihood ratio test were used to extract a reduced model from the multiple model. In these two steps, the adjusted OR was obtained for each variable and the main interpretation was performed based on the final model.

## Results

### Demographic characteristics of women with BC

This study was conducted on 210 women with BC undergoing chemotherapy. The mean and standard deviation of age and number of breast screening referrals were 45.1 ± 10.8 and 3.11 ± 3.22, respectively. Among the respondents, 76.2% were married and the maximum educational level attained by the majority of the respondents was elementary school completion (33.3%). The participants’ age ranged from 20 to 77 years. Of the participants, 83.3% were housewives; 78.6% had ordinary insurance; 80% did not have a family history of BC; the health literacy of 70% was inadequate; and the median (IQR) number of children was 3 (1 to 4) (Table 1).

**Table 1:** Demographic characteristics of women with BC.

Variable	Number	Percentage
Marital status		
Single	29	13.8
Married	160	76.2
Divorced	8	3.8
Widow	13	6.2
Education level		
Illiterate	36	17.1
Elementary	70	33.3
Primary high school	25	11.9
Secondary high school	11	5.2
Diploma	38	18.1
University	30	14.3
Occupation		
Housewife	175	83.3
Employee	25	11.9
Self-Employee	2	1.0
Other	8	3.8
Insurance coverage		
None	12	5.7
Basic insurance	165	78.6
Supplementary insurance	33	15.7
Health literacy		
Inadequate	147	70
Relatively inadequate	45	21.4
Sufficient	16	7.6
Excellent	2	1.0
Family history of breast cancer		
Yes	42	20
No	168	80
	Mean ± SD	
Age	45.1 ± 10.8	
Number of visits for breast screening examination	3.11 ± 3.22	
	Median (25–75)^¥^	
Number of children	3 (1–4)	

¥: Median (interquartile 25–75).

### The distribution of the mean scores of the subscales and the overall health literacy in the participating women with BC

The mean and standard deviation of overall health literacy score was 40.35 ± 19.01 in women with BC in the south of Iran, indicating insufficient health literacy in this group. The maximum and minimum health literacy scores belonged to the domains of understanding and perception (24.22 ± 7.54) and reading (12.09 ± 4.61), respectively (Table 2).

**Table 2:** The distribution of the mean scores of the subscales and the overall health literacy in the participating women with BC.

Subscales	Mean	SD	Min	Max
Reading	12.09	4.61	4.00	20.00
Access	21.41	6.86	7.00	35.00
Understanding and perception	24.22	7.54	9.00	45.00
Evaluation and judgment	14.04	4.47	5.00	25.00
Decision making and behavior	17.12	7.85	9.00	43.00
Overall	40.35	19.01	0.00	87.5

SD: standard deviation; Min: minimum; Max: maximum.

### The relationship between the predictor variables and health literacy in the participating women with BC using logistic regression models

Univariate logistic regression shows that age of participating women with BC > 45 years is significantly associated with their health literacy (*p* = 0.026). Hence, with every one unit increase in the women’s age > 45 years, the probability of better health literacy will increase by 0.53 times. Furthermore, as per the backward stepwise multiple logistic regression model, educational level (university) as well as supplementary insurance coverage are reported to be the prime predictor of health literacy among women with BC. After adjusting for all the predictor variables, educational level (university) and supplementary insurance coverage of the women are significantly (*p* < 0.001) associated with health literacy with odds ratio of 4.41 and 5.83 (Table 3).

**Table 3:** The relationship between the predictor variables and health literacy in the participating women with BC using logistic regression models.

Variables	Univariate logistic regression model		Multiple logistic regression model		Backward stepwise multiple logistic regression model			
	OR	*p*-value	Adjusted OR	*p*-value	Adjusted OR	95% CI for OR		*p*-value
						Upper	Lower	
Age > 45 years	0.53	0.026	0.62	0.139	–	–	–	–
Marital status	0.85	0.474	0.95	0.873	–	–	–	–
Number of children	0.92	0.207	0.97	0.770	–	–	–	–
Occupation	1.49	0.076	0.80	0.495	–	–	–	–
Number of visits for breast screening examination	1.03	0.451	1.05	0.311	–	–	–	–
Positive family history of BC	0.88	0.730	0.49	0.092	–	–	–	–
Education level (university)	5.47	<0.001	5.14	0.009	4.41	1.54	12.57	0.005
Insurance coverage (supplementary)	6.41	<0.001	6.82	<0.001	5.83	2.17	15.62	<0.001

OR: odds ratio; CI: confidence interval.

## Discussion

Cancer treatment is a stressful experience and patients need information to help with decision-making, treatment, and better understanding their disease. Therefore, health literacy has an undeniable effect on the process of treating diseases, especially chronic ones (19). This study aimed to determine the level of health literacy among women with breast cancer undergoing chemotherapy in teaching hospitals affiliated with Zahedan University of Medical Sciences in 2020 and examine the related factors. Based on the results, the mean total score of health literacy was 40.35 in the BC patients, indicating insufficient health literacy. The present findings are consistent with the results reported by William in the United States and Ghanbari in Iran (29, 30), which also showed that health literacy was insufficient in women with BC undergoing chemotherapy. Ghanbari et al. reported a health literacy rate of 54.6% in their study (31). In a study conducted in Sistan and Baluchestan Province of Iran, Izadi et al. concluded that only 32% of the study population was sufficiently literate (32), while in a study assessing the health literacy level among fertile Iranian women with BC, Haghighi et al. found a high level of health literacy, which is inconsistent with the present findings (33). One of the reasons for this discrepancy may be attributed to the study samples and the role of pregnancy.

Based on the results, with every one-unit increase in the women’s age > 45 years, the probability of better health literacy will increase by 0.53 times. In line with the findings of the present study, Rahmanian et al. reported that with each year of age, diabetic patients’ health literacy level increased (34). Other studies on the association between age and different levels of health literacy have found with age increasing, information on various health topics increases and people due to longer exposure time with the disease, gain more and more appropriate experiences (35–38).

Among the factors affecting the level of health literacy in women with breast cancer, there was a significant relationship between health literacy and the level of education. Patients with university education had a higher mean health literacy score, which confirms the direct and significant effect of education on health literacy. Khosravi et al. determined the level of health literacy in diabetic patients and its contributing factors and showed that there is a positive and significant relationship between the level of education and health literacy, which is in line with the present findings (39). Moreover, in a study conducted in the south of Iran, Saberipour et al. concluded that there is a positive and significant relationship between the health literacy of inpatients and their level of education (40). In a study in Taiwan, Lee et al. (36) also found that there is a direct and significant relationship between patients’ health literacy and their level of education. Ozen et al., in a study aimed at determining the factors affecting the health literacy of nursing students in Turkey, concluded fourth-year students had higher health literacy than ones in other years, indicating the education effect on health literacy (41). All these studies indicate that people with higher education have greater health literacy and understand and use health information and instructions better. Furthermore, patients with a lower level of education might enjoy a lower level of health literacy and have trouble understanding and using health information, understanding the use and method of administration of medications, and understanding medical orders; they therefore need further training in this regard.

A direct and significant relationship was found between the type of health insurance and the level of health literacy; those with supplementary health insurance had the highest level of health literacy compared to the other participants (those who had no health insurance or had basic insurance). Calvo et al. (42) determined the relationship between the level of health literacy and the quality of healthcare in the US. In line with the present findings, they concluded that the level of education is one of the factors affecting the level of health literacy (42). Kino et al. (43) determined the relationship between medical insurance services and health literacy in different social strata in the US. They concluded that health literacy has a direct and significant association only with dental services (43). In other words, dental services make up the majority of medical services covered by supplementary health insurance. In Iran, too, supplementary health insurance coverage focuses on dental and optometry services and prescription glasses. According to statistics published by Iranian cancer studies, only 30% of the services provided to cancer patients are covered by insurance, which belongs to people who have supplementary insurance. For example, Jabbari et al. (44) showed that the mean direct non-medical cost paid out of pocket per month was $89.9 ± $11.9 USD and the mean direct medical cost per month was $738.9 ± $55.85 USD. The total cost paid by the patients was $828.4 ± 59.92 USD per month for patients with BC and concluded that insurance companies must fully cover healthcare for cancer patients in order to prevent the families from suffering from the cost of living (44). In the study by Longo et al. (45) breast cancer patients spent a larger amount of monthly out-of-pocket payments than those with colorectal, lung, and prostate cancers. In addition, patients with breast cancer also suffered from a larger financial burden, as compared with other patients (31% vs. 17%) (45). Therefore, insurance services affect people’s health literacy, and those who have better insurance coverage benefit from more preventive services and therefore have higher health literacy.

Finally, based on the results of the multivariate regression analysis, no significant relationship was found between the health literacy of people with breast cancer and age, marital status, the number of children, occupation, the number of breast cancer screenings, and family history of breast cancer (*p* > 0.05).

Health literacy plays a crucial role in the journey of women with breast cancer, influencing their ability to navigate the complex web of health information, make informed decisions, and effectively communicate with healthcare providers (46). Breast cancer is a disease that often requires patients to digest vast amounts of medical information, from understanding treatment options to managing side effects and interpreting diagnostic results. For women with limited health literacy, comprehending these intricacies can be challenging, leading to increased anxiety and confusion. This knowledge gap may hinder their ability to actively engage in discussions with their healthcare team, ask pertinent questions, and make informed choices about their treatment (47). As a result, improving health literacy among women with breast cancer is essential to enhance their overall health outcomes and quality of life.

Effective health communication is a critical component of supporting women with breast cancer, and it goes hand in hand with health literacy (48). When healthcare providers communicate clearly, using plain language and visuals when necessary, they can bridge the gap between their medical expertise and the patient’s understanding. Additionally, tailored communication strategies that consider individual health literacy levels can help empower patients, enabling them to actively participate in shared decision-making (49). By fostering a supportive and open dialogue, healthcare professionals can address concerns, alleviate fears, and provide emotional support throughout the breast cancer journey. Therefore, the association between health literacy and health communication is symbiotic, where improved health literacy enhances the effectiveness of health communication, ultimately leading to better breast cancer outcomes for women (50).

More extensive and multi-centered studies are recommended for a deep and accurate examination of the relationship between the factors affecting the level of health literacy in breast cancer patients and to confirm the results of the present study.

### Limitations

This study has some limitations. First, it was cross-sectional in design and the participants were asked to complete the questionnaire themselves, and a self-administered questionnaire could be biased. Second, since this research has been performed only on patients in two teaching hospitals affiliated to Zahedan University of Medical Sciences, the generalization of its results to other centers and settings needs to be carried out with caution. Future studies are recommended to focus on the same topic but in different regions of Iran.

## Conclusion

As the prevalence of BC is increasing in Iran and the treatments offered to this group of patients put a lot of financial pressure on the healthcare system, more research should be performed on preventive behaviors of breast cancer. Designing health promotion programs should be one of the main targets of the national healthcare system. As one of the main factors involved in the promotion of health, health literacy is a prerequisite of living in today’s modern societies (4). Health literacy can have a positive effect on people’s health status by empowering them to make good health decisions. People with good levels of health literacy are more aware of their need for preventive measures that help maintain or promote their health.

## Data availability statement

The original contributions presented in the study are included in the article/Supplementary material, further inquiries can be directed to the corresponding author.

## Ethics statement

The studies involving humans were approved by Ethic Committee of Zahedan University of Medical Sciences (IR.ZAUMS.REC.1399.242). The studies were conducted in accordance with the local legislation and institutional requirements. The participants provided their written informed consent to participate in this study.

## Author contributions

RS, MH, SS, AS, and FK conceived and designed the study and draft the manuscript. RS, MH, and SS collected, input, and checked the data. HB analyzed the data and submitted the manuscript. HB and SS revised the manuscript. All authors contributed to the article and approved the submitted version.

## Supplementary material

Supplementary Table 1 The Health literacy questionnaire of Iranian women with BC.
