# Supplementary material for: The health literacy level and its related factors in Iranian women with breast cancer undergoing chemotherapy
Source: Front Public Health. 2023 Sep 28;11:1150148. doi: 10.3389/fpubh.2023.1150148 (PMC10568019; doi:10.3389/fpubh.2023.1150148)
Supplement: Supplementary Table 1 — The Health literacy questionnaire of Iranian women with BC. [file Table_1.DOCX]

**The Health literacy questionnaire of Iranian women with BC**

| Dimensions | Items | Sentences | Quite easy | Easy | Neither easy nor difficult | Hard | Quite hard |
| --- | --- | --- | --- | --- | --- | --- | --- |
|  |  |  | 5 | 4 | 3 | 2 | 1 |
| **Reading** | 1 | Reading Persian educational materials about breast cancer in educational booklets and pamphlets |  |  |  |  |  |
|  | 2 | Reading the written instructions that the medical and health staff sometimes give me regarding breast cancer |  |  |  |  |  |
|  | 3 | Reading the consent form before any surgical and medical procedures |  |  |  |  |  |
|  | 4 | Reading medical forms such as the patient admission form in treatment departments (such as chemotherapy and radiation therapy) |  |  |  |  |  |
| **Access** | 5 | Access to information related to breast cancer from written sources such as books, booklets and educational pamphlets |  |  |  |  |  |
|  | 6 | Access to information about side effects of chemotherapy, hormone therapy, radiation therapy, surgery, etc. from different sources |  |  |  |  |  |
|  | 7 | Access to information about treatment-related care such as healthy diet, not smoking, physical activity, etc. from various sources |  |  |  |  |  |
|  | 8 | Access to various information related to follow-up examinations after chemotherapy, hormone therapy, radiation therapy, surgery, etc. from various sources |  |  |  |  |  |
|  | 9 | Access to information about healthy nutrition and its benefits from various sources |  |  |  |  |  |
|  | 10 | Access to information about smoking and its harms from different sources |  |  |  |  |  |
|  | 11 | Access to information about breast reconstruction after breast removal surgery from different sources |  |  |  |  |  |
| **Understanding and perception** | 12 | Understanding information related to breast cancer from written sources such as books, booklets and educational pamphlets |  |  |  |  |  |
|  | 13 | Understanding information about breast cancer from radio and television |  |  |  |  |  |
|  | 14 | Understanding information related to treatment methods such as surgery and drug therapy from written sources of books, booklets and educational pamphlets |  |  |  |  |  |
|  | 15 | Understanding information related to treatment complications (chemotherapy, hormone therapy, radiation therapy, surgery, etc.) from different sources |  |  |  |  |  |
|  | 16 | Understanding information about treatment-related cares such as healthy diet, not smoking, physical activity, etc. from different sources |  |  |  |  |  |
|  | 17 | Understanding information related to follow-up examinations after chemotherapy, hormone therapy, radiation therapy, surgery, etc. from different sources |  |  |  |  |  |
|  | 18 | Understanding information about healthy nutrition and its benefits from different sources |  |  |  |  |  |
|  | 19 | Understanding information about smoking and its harm from different sources |  |  |  |  |  |
|  | 20 | Understanding information related to breast reconstruction after breast removal surgery from different sources |  |  |  |  |  |
| **Evaluation and judgement** | 21 | Assessing the accuracy of radio and television information about breast cancer |  |  |  |  |  |
|  | 22 | Assessing the accuracy of the information provided by my friends or relatives about breast cancer and my health |  |  |  |  |  |
|  | 23 | Assessing the accuracy of the information about preventive factors of breast cancer |  |  |  |  |  |
|  | 24 | Assessing the accuracy of the information provided by the doctor about the courses and progress or improvement of the disease |  |  |  |  |  |
|  | 25 | Assessing the accuracy of the information provided by the doctor about the advantages and disadvantages of different treatment methods (surgery, radiation therapy, hormone therapy, etc.) |  |  |  |  |  |
| **Decision making and behavior** | 26 | I do mammography to follow up my disease, according to the doctor's order |  |  |  |  |  |
|  | 27 | If the doctor prescribes medicine, I take it as prescribed |  |  |  |  |  |
|  | 28 | I avoid smoking. |  |  |  |  |  |
|  | 29 | I follow the nutritional principles according to the nutritional recommendations of the treatment team (doctor, dietitian, etc.) |  |  |  |  |  |
|  | 30 | I go to the doctor, if See any kind of bleeding and discharge from the nipple |  |  |  |  |  |
|  | 31 | I go to the doctor, if See breast pain |  |  |  |  |  |
|  | 32 | I go to the doctor, if See any kind of changes in the breast tissue |  |  |  |  |  |
|  | 33 | I follow the recommendation of my specialist doctor to do chemotherapy, radiotherapy and other treatments |  |  |  |  |  |
|  | 34 | If the doctor asks for some types of imaging to predict the spread of my disease, I will do it |  |  |  |  |  |
